# Reliability and group differences in quantitative cervicothoracic measures among individuals with and without chronic neck pain

**DOI:** 10.1186/1471-2474-13-215

**Published:** 2012-10-31

**Authors:** Bahar Shahidi, Cynthia L Johnson, Douglas Curran-Everett, Katrina S Maluf

**Affiliations:** 1 Physical Therapy Program, University of Colorado Anschutz Medical Campus, Aurora, CO, USA; 2 Physical Therapy Program, University of Colorado Anschutz Medical Campus, Aurora, CO, USA; 3 Division of Biostatistics and Bioinformatics, National Jewish Health, Denver, CO, USA; 4 Physical Therapy Program, University of Colorado Anschutz Medical Campus, Aurora, CO, USA; 5 Department of Physical Medicine & Rehabilitation Physical Therapy Program, University of Colorado Anschutz Medical Campus, MS C244 Education 2 South. Bldg #L28 13121 E. 17th Ave, Room 3108, Aurora, CO, 80045, USA

**Keywords:** Scapular strength, Reliability, Neck pain, Cervical, Thoracic

## Abstract

**Background:**

Clinicians frequently rely on subjective categorization of impairments in mobility, strength, and endurance for clinical decision-making; however, these assessments are often unreliable and lack sensitivity to change. The objective of this study was to determine the inter-rater reliability, minimum detectable change (MDC), and group differences in quantitative cervicothoracic measures for individuals with and without chronic neck pain (NP).

**Methods:**

Nineteen individuals with NP and 20 healthy controls participated in this case control study. Two physical therapists performed a 30-minute examination on separate days. A handheld dynamometer, gravity inclinometer, ruler, and stopwatch were used to quantify cervical range of motion (ROM), cervical muscle strength and endurance, and scapulothoracic muscle length and strength, respectively.

**Results:**

Intraclass correlation coefficients for inter-rater reliability were significantly greater than zero for most impairment measures, with point estimates ranging from 0.45 to 0.93. The NP group exhibited reduced cervical ROM (P ≤ 0.012) and muscle strength (P ≤ 0.038) in most movement directions, reduced cervical extensor endurance (P = 0.029), and reduced rhomboid and middle trapezius muscle strength (P ≤ 0.049).

**Conclusions:**

Results demonstrate the feasibility of obtaining objective cervicothoracic impairment measures with acceptable inter-rater agreement across time. The clinical utility of these measures is supported by evidence of impaired mobility, strength, and endurance among patients with NP, with corresponding MDC values that can help establish benchmarks for clinically significant change.

## Background

Neck pain is a condition that is commonly treated by health care professionals. It has been estimated that the annual prevalence of neck pain in the general population is 30-50%, with the prevalence of activity limitations due to neck pain ranging between 11-14% [1,2]. Neck pain can be categorized based on the duration of symptoms as acute (less than 7 days), sub-acute (between 7 days and 3 months), or chronic (greater than 3 months) [3]. Whereas the majority of individuals who experience acute symptoms do not seek professional care, chronic neck pain has a prolonged negative impact on health and health care expenditures [4].

Several studies have investigated the reliability of cervical impairment measures such as strength [5,6], endurance [5,6], and range of motion [6,7] among individuals with neck pain. As reviewed by Nordin and colleagues [6], a large number of these studies examined patients with acute or whiplash associated neck pain, in which measurement reliability may be reduced by limited tolerance for maximal performance testing after an acute injury. Moreover, the natural time course of symptom resolution is more predictable in patients with acute compared to chronic neck pain [3], which ultimately limits the ability to generalize findings from an acute pain population to patients who are experiencing chronic symptoms. Given that chronic symptoms tend to fluctuate over time, it is important to establish the between-day reliability and minimum detectable change (MDC) for cervical impairment measures so that clinicians can identify meaningful improvement in patients treated for chronic neck pain. Finally, the reliability of cervical impairment measures has most often been examined within a single session [8-11] limiting the ability to generalize these findings to a clinical setting where impairments are typically reassessed days or weeks apart, often by different therapists.

In addition to establishing the reliability and MDC of cervical impairment measures, it is also important to identify whether there are systematic differences in the range of values typically observed among individuals with and without chronic neck pain. Normative data from healthy individuals can help identify which impairments should be targeted for assessment in patients with neck pain, and provide an empirical basis for judging the severity of impairments for individual patients.

In the absence of quantitative assessment tools for neck pain that are both valid and feasible, clinicians often rely on the subjective categorization of musculoskeletal impairments. For example, muscle length is often categorized as “within normal limits” or “short” as compared to the contralateral limb [8,12]. Similarly, muscle strength is often categorized on an ordinal scale based on manual muscle testing (MMT) as described by Kendall and McCreary [12]. Although the MMT scale is commonly used in clinical practice, it lacks sensitivity to detect improvements in strength among individuals whose muscles are neurologically intact and able to withstand a relatively high magnitude of manual resistance [13]. Recent studies have demonstrated the utility of hand held dynamometry (HHD) as a robust alternative to MMT which shows acceptable reliability for a variety of different tests of isometric strength across several muscle groups [9,13]. Although scapulothoracic muscles such as the rhomboids, middle trapezius, and lower trapezius are thought to contribute to postural stability of the cervical spine and reduce biomechanical loading of cervicoscapular musculature [14,15], we are aware of only one study that has investigated the use of HHD to measure scapulothoracic muscle strength in individuals with neck pain [16]. It is currently not known whether scapulothoracic muscle strength is impaired in patients with chronic neck pain compared to healthy individuals, or whether the strength of these muscles can be reliably assessed over time.

Whereas numerous studies have reported the intra- and inter-rater reliability of cervical impairment measures, systematic differences between individuals with and without neck pain and the MDC required to detect clinically significant improvement over time have not been established for the majority of these measures. This limits the ability of clinicians to identify meaningful thresholds of cervical impairment, and to track quantitative changes in these impairments following treatment. The reliability and validity of scapulothoracic impairment measures are also not known, despite being commonly addressed in interventions for chronic neck pain. Therefore, the purpose of this study was to assess the inter-rater reliability, MDC, and group differences in quantitative cervical and scapulothoracic impairment measures among individuals with and without chronic neck pain.

## Methods

### Participants

Participants between the ages of 18 and 65 years were recruited from a university medical campus and surrounding community. The healthy control group included 20 participants who reported no history of neck pain in the last year, and had a Neck Disability Index (NDI) score of less than 5 points at the time of enrollment. This cutoff was selected based on the classification scheme of Vernon and Mior [17] in which fewer than 5 points indicates no neck related disability. The neck pain group included 19 participants with a primary complaint of neck pain for at least 3 months prior to enrollment, and an NDI score of greater than 5 points. In order to generalize the study findings to individuals with a significant restriction of functional activities due to neck pain, only those individuals who met the Neck Pain Task Force [18] definition of grade I or II interfering neck pain with unilateral or bilateral symptoms located between the superior nuchal line and the superior spine of the scapula were included. The neck pain and control groups were matched based on sex, age, and body mass index. Ninety percent of the individuals in each group were right hand dominant. Exclusion criteria included any reported history of central nervous system impairment, signs or symptoms consistent with cervical nerve root compression or other non-musculoskeletal sources of pain, and prior surgery involving the cervical or thoracic spine. Although the majority of participants reported prior episodes of professional or self-treatment of neck symptoms, participants currently under the care of a health care professional for the treatment of neck pain were excluded due to the confounding effects of concurrent treatment on symptom severity. Medications were documented for all participants at the time of testing, and did not change across test sessions. All participants provided written informed consent prior to enrollment, and all study procedures were approved by the local Institutional Review Board.

### Examiners

Two licensed physical therapists served as the examiners for this study. Both therapists participated in three, one-hour training sessions to standardize the examination procedures. Rater One had 2 years of clinical experience in an outpatient orthopedic setting, and was enrolled in a certification program for manual therapy. Rater Two had 23 years of clinical experience, with post entry-level certification in hand therapy.

### Procedures

A repeated measures design was used to separately assess the inter-rater reliability of cervical and scapulothoracic impairment measures for individuals with and without chronic neck pain. Participants underwent one physical examination by each therapist on two different days in random order. As a safety precaution, therapists were not blinded to group assignment so that any worsening of symptoms could be monitored during the examination. However, all participants were instructed not to provide any clinical information or cues not related to the examination with the therapist prior to each session. Therapists remained blinded to the other rater's findings between examinations. Tests were performed in the same order for each examination, and tests that required multiple measurements by the same rater were performed consecutively with a 30-60s break between measurements. The examination included measures of cervical range of motion, strength, and endurance, as well as scapulothoracic muscle length and strength. Each examination lasted approximately 30 minutes. Sessions were performed at approximately the same time of day for each participant, with an average (SD) of 9 (4) days (range 3–14 days) between sessions.

### Cervical active range of motion

Active cervical flexion, extension, and side bending range of motion was assessed using a gravity inclinometer (Medical Research Limited, Leer, U.K) with the participant sitting upright according to procedures described by Cleland et al [8]. Active cervical rotation range of motion was assessed using the same inclinometer with the participant lying supine as previously described by Hoving et al [10]. Three trials were performed in each direction to assess the intra-rater reliability of within-session measurements, and the average of the three measurements from each rater was used to assess the inter-rater reliability between sessions.

### Cervical muscle strength

**Figure 1:**
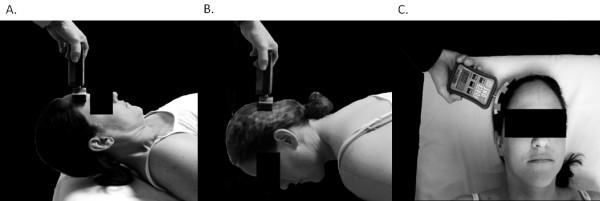
**Cervical muscle isometric strength test positions for flexion** (**A**), **extension** (**B**), **and side bending** (**C**).

Cervical flexion, extension, and side bending strength was assessed using a HHD (FPIX 100kg load cell, Wagner Instruments, Greenwich, CT). For isometric flexion strength, participants were positioned in supine and asked to hold their head in approximately 30 degrees of flexion with the chin tucked while the examiner applied a force into the direction of cervical extension with the HHD centered on the forehead [19] (Figure 1a). For isometric extension strength, participants were positioned in prone with the shoulders supported at the edge of the examination table and the head held against gravity just beyond the edge of the table. Participants were asked to hold their head in a neutral position while the examiner provided a force into the direction of cervical flexion with the HHD centered on the back of the head (Figure 1b). Based on pilot testing, which revealed poor trunk and head stabilization in sitting and side lying during the assessment of isometric side bending strength, this outcome was assessed in supine. Participants were instructed to maintain a neutral head position with the back of the head resting against the examination table, while the examiner provided a force into the direction of side bending with the HHD centered on the contralateral side of the head (Figure 1c). Participants were stabilized in supine using a 4-inch velcro strap placed across the chest at the level of the sixth thoracic vertebrae (T6) and across the pelvis at the level of the anterior superior iliac spine to prevent movement of the upper body as force was applied to the head. For all isometric force measurements, manual resistance was applied at a rate of approximately 3 kg∙F/s and the maximum force recorded by the dynamometer while the participant was still able to maintain the test position was considered the maximum isometric force. Only one maximum strength test was performed in each direction to minimize the potential for reduced force output with increased cervical pain due to repeated testing. Single trial results were used to assess the inter-rater reliability of isometric cervical strength measurements between sessions.

### Cervical muscle endurance

**Figure 2:**
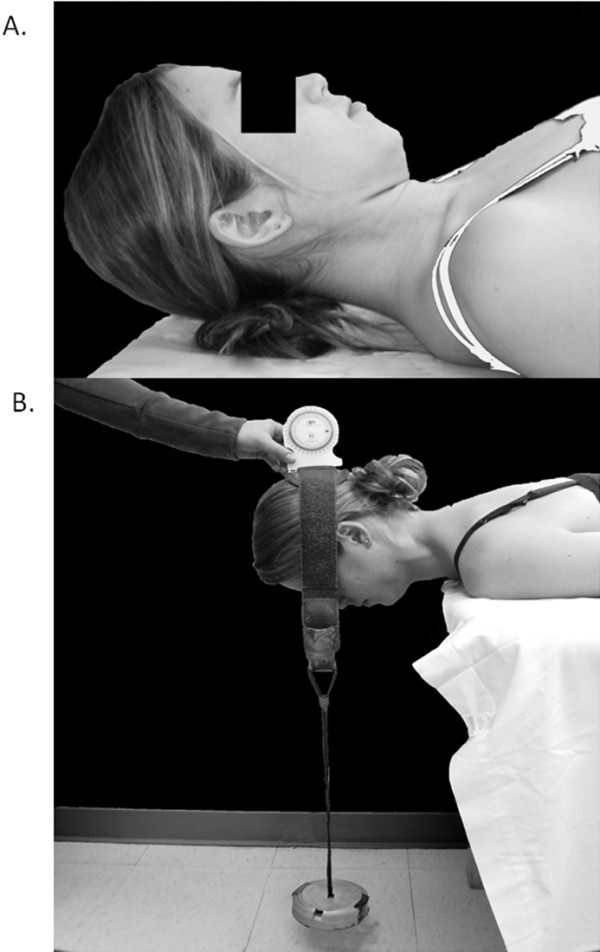
**Cervical muscle isometric endurance test positions for flexion** (**A**) **and extension** (**B**).

The isometric endurance of cervical flexors was assessed as described by Cleland et al [8] with participants positioned in supine, the upper cervical spine flexed, and the head held approximately 1 inch above the examination table (Figure 2a). Participants were asked to maintain this head position for as long as possible. The endurance test was terminated when participants were no longer able to keep their head from touching the table, or when upper cervical flexion could not be maintained. Loss of craniocervical flexion was assessed by observing the position of the chin and the skin fold produced posterior to the mandible when the head was placed in the test position. A change in the thickness of this skin fold or visible motion of the chin was interpreted as a loss of craniocervical flexion, resulting in termination of the endurance test.

The isometric endurance of cervical extensors was assessed as described by Edmondston et al [20] with participants positioned in prone, the head held in a neutral position just beyond the edge of the examination table, and both arms at the sides with a 4-inch stabilization strap across the thoracic spine at the level of T6. A 2-inch velcro band was secured around the head with a fluid inclinometer placed over the occiput. A 2 kg weight was suspended from the headband, and participants were asked to support the weight while maintaining a neutral head position for as long as possible (Figure 2b). The endurance test was terminated when the position of the head changed by more than 5 degrees from the horizontal, or a maximum endurance time of 5 minutes was achieved. Endurance time was measured using a handheld stopwatch. One trial was performed for each direction to minimize the potential for increased cervical pain with repeated testing, and this value was used to assess the inter-rater reliability of cervical muscle endurance between sessions.

### Scapulothoracic muscle strength

**Figure 3:**
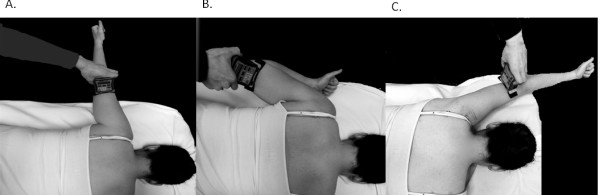
**Scapulothoracic muscle isometric strength test positions for middle trapezius** (**A**), **rhomboid** (**B**), **and lower trapezius** (**C**).

Isometric strength of the middle trapezius, rhomboids, and lower trapezius muscles was tested bilaterally with participants lying prone and the HHD placed one inch proximal to the lateral epicondyle of the elbow. Participants were stabilized in this position using 4-inch velcro straps placed across the pelvis at the level of the posterior superior iliac spine and across the thighs just proximal to the knee joint. Test positions for each muscle were performed according to descriptions provided by Kendall [12] (Figure 3a-c). Manual resistance was applied at a rate of approximately 3 kg∙F/s, and the maximum force recorded by the dynamometer while the participant was still able to maintain the test position was considered the maximum isometric force. Pilot testing revealed that three maximal effort trials of the scapulothoracic musculature were well tolerated by participants without an increase in primary cervical symptoms. Therefore, three trials were performed for each muscle with at least 60 seconds rest between trials to assess the intra-rater reliability of within-session measurements. The average of the three trials for each rater was used to assess the inter-rater reliability of isometric scapulothoracic muscle strength between sessions.

### Scapulothoracic muscle length

**Figure 4:**
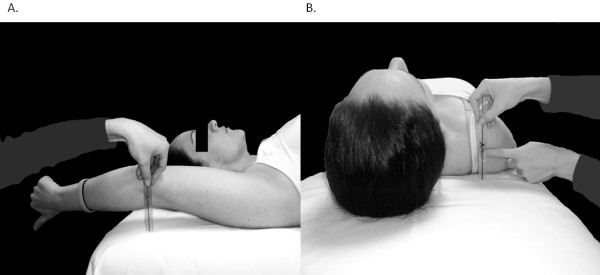
**Scapulothoracic muscle length test positions for latissimus dorsi** (**A**) **and pectoralis minor** (**B**).

Length of the latissimus dorsi and pectoralis minor muscles was estimated bilaterally using a standard ruler in the test positions described by Kendall [12] and others [8]. These muscles were selected for length testing based on their suggested effects on alignment and movement of the scapulothoracic region [15] and for comparison to a previous study [8]. Latissimus dorsi length was estimated as the distance from the lateral epicondyle to the surface of the examination table with the upper arms positioned in maximal flexion as participants lay supine with their knees bent and the lumbar spine in full contact with the table (Figure 4a). Resting length of the pectoralis minor was estimated as the distance from the posterolateral aspect of the acromion to the surface of the examination table with participants resting in supine and both arms at the sides (Figure 4b). Only one measurement was performed for each muscle to avoid changes in muscle length that can occur with repeated movements of viscoelastic tissues. Single trial results were used to assess the inter-rater reliability of muscle length measurements between sessions.

### Data analysis

Descriptive statistics were calculated as means and standard deviations (SD). After confirming a normal distribution of all impairment measures using the Komologrov-Smirnoff test, point estimates and 95% confidence intervals (CI_95_) were calculated using intraclass correlation coefficients (ICC) to determine the intra-rater reliability of cervical range of motion and scapulothoracic strength measures obtained by each rater within the same session based on a two way mixed model for absolute agreement (ICC(3,1)). Inter-rater reliability across the two test sessions was calculated using a two way random effects model for absolute agreement. The average of three trials for cervical range of motion and scapulothoracic strength measures was assessed using ICC(2,3), whereas single measurements of cervical muscle strength, cervical muscle endurance, and scapulothoracic muscle length were assessed using ICC(2,1). The level of reliability is qualitatively described in the text according to Landis and Koch [21] with ICC values of 0.0-0.20 indicating slight agreement, 0.21-0.40 indicating fair agreement, 0.41-0.60 indicating moderate agreement, 0.61-0.80 indicating substantial agreement, and 0.81-1.00 indicating almost perfect agreement. ICC values were considered to indicate no significant agreement if the 95% CI included zero.

To determine the smallest change in each impairment measure that can confidently be considered to exceed measurement error at a 95% confidence level, the Minimum Detectable Change (MDC) was calculated according to the following formula [22]: MDC = 1.96 · √2 · SD · √(1 − test–retest reliability coefficient). MDC values were calculated separately for the neck pain and healthy control groups. Independent t-tests were used to compare cervical and scapulothoracic impairment measures between the neck pain and healthy groups, with each outcome computed as the average value across both raters. A 3-way ANOVA (group х rater х order) with repeated measures on the latter two factors was performed to identify any effects of the rater (Rater 1 vs. Rater 2) or test order (session 1 vs. session 2) on impairment measures. All statistical analyses were performed using SPSS software v.16.0.1 (Chicago, IL, USA).

## Results

Descriptive characteristics of the study population are summarized in Table 1. There were no significant differences between the neck pain and healthy control groups, with the exception of NDI scores which were significantly greater for individuals with neck pain (P<0.001). Intra-rater reliability coefficients for cervical range of motion across all movement directions were almost perfect with ICC values ranging from 0.95 (CI_95_ 0.87-0.98) to 0.98 (CI_95_ 0.94-0.99) in the healthy group and from 0.94 (CI_95_ 0.85-0.98) to 0.98 (CI_95_ 0.95-0.99) in the neck pain group for Rater 1. Similarly, cervical range of motion intra-rater reliability coefficients for Rater 2 ranged from 0.50 (CI_95_ 0.22-0.73) to 0.97 (CI_95_ 0.93-0.99) in the healthy group, and from 0.95 (CI_95_ 0.89-0.98) to 0.99 (CI_95_ 0.98-0.99) in the neck pain group. Intra-rater reliability coefficients for scapulothoracic strength across all muscles were substantial to almost perfect, with ICC values ranging from 0.92 (CI_95_ 0.84-0.97) to 0.94 (CI_95_ 0.88-0.97) and 0.84 (CI_95_ 0.70-0.93) to 0.93 (CI_95_ 0.86-0.97) for Rater One, and from 0.80 (CI_95_ 0.64-0.91) to 0.95 (CI_95_ 0.90-0.98) and 0.71 (CI_95_ 0.50-0.87) to 0.92 (CI_95_ 0.84-0.97) for Rater Two in the healthy and neck pain groups, respectively.

**Table 1:** Subject characteristics

	**Healthy** (**N** =** 20**)	**Neck pain** (**N **= **19**)	**P value**
Sex (M:F)	10:10	10:9	0.87
Age (yrs)	34.0 (10.4)	34.9 (9.9)	0.93
Height (m)	1.76 (0.09)	1.73 (0.10)	0.38
Weight (kg)	65.61 (20.85)	72.13 (21.37)	0.33
NDI (points)	0.6 (1.2)	14.4 (7.3)	< 0.001

Values are mean (SD) unless otherwise indicated. Abbreviations: M=male; F=female; NDI=Neck Disability Index.

Table 2 provides the group averages calculated separately for each rater, along with the inter-rater reliability coefficients and MDC values for all cervicoscapular impairment measures. There was no significant main effect of test order on any outcome (P ≥ 0.11). Significant rater effects (P < 0.05) were observed for a small number of outcomes in Table 2, with the less experienced rater (Rater 1) recording higher values of cervical strength and lower values of latissimus dorsi length on average. Reliability coefficients for cervical range of motion ranged from moderate to substantial agreement in both healthy (ICC = 0.45 to 0.79) and neck pain (ICC = 0.47 to 0.78) groups, with MDC values ranging from 5 to 15 degrees for the healthy group and from 9 to 21 degrees for the neck pain group. The reliability of cervical strength measures ranged from substantial to almost perfect agreement for the healthy group (ICC = 0.67 to 0.85), and from fair to substantial agreement for the neck pain group (ICC= 0.39 to 0.72). MDC values for cervical strength ranged from 4.4 to 9.5 kg∙F and from 7.2 to 12.5 kg∙F for the healthy and neck pain groups, respectively. Cervical endurance measures demonstrated substantial agreement for flexion (ICC (CI_95_) = 0.72 (0.42-0.88); MDC = 34.1s), but no significant agreement for extension (ICC (CI_95_) = 0.03 (−0.50-0.52); MDC = 178.3s) among healthy individuals. In contrast, cervical endurance measures in the neck pain group demonstrated no significant agreement for flexion (ICC (CI_95_) = 0.40 (−0.07-0.72); MDC = 27.8s), but almost perfect agreement for extension (ICC (CI_95_) = 0.83 (0.61-0.93); MDC = 110.2s). Reliability coefficients for scapulothoracic muscle strength ranged from moderate to almost perfect in the healthy group (ICC = 0.58 to 0.88; MDC = 4.2-11.6 kg∙F), and from fair to substantial in the neck pain group (ICC = 0.33 to 0.78; MDC = 3.9-9.2 kg∙F). Reliability coefficients for muscle length were generally higher for the healthy group, in which ICC values ranged from 0.40 to 0.93 (MDC range = 1.6 to 4.2 cm), compared to the neck pain group in which ICC values ranged from 0.19 to 0.82 (MDC range = 1.4 to 7.6 cm).

**Table 2:** Average values, reliability and minimum detectable change for cervical and scapulothoracic impairment measures

**Impairment measure**	**Healthy**			**Neck pain**		
	**Rater 1** (**Ave**, **SD**)	**Rater 2** (**Ave**, **SD**)	**ICC** (**95**% **CI**); **MDC**	**Rater 1** (**Ave**, **SD**)	**Rater 2** (**Ave**, **SD**)	**ICC** (**95**% **CI**) ; **MDC**
***Cervical AROM*** (***degrees***)						
Flexion	61(8)	58(8)	0.45 (0.06-0.74); 14	48(11)	48(13)	0.69 (0.36-0.87); 16
Extension	70(14)	67(11)	0.79 (0.54-0.91); 15	56(13)	56(13)	0.78 (0.50-0.91); 16
R. Side bend	42(7)	44(8)	0.58 (0.21-0.81); 12	39(7)	43(6)	0.47 (0.06-0.75); 12
L. Side bend	46(8)	45(7)	0.79 (0.54-0.91); 9	39(7)	39(6)	0.68 (0.34-0.87); 9
R. Rotation*	87(9)	85(8)	0.69 (0.37-0.87); 12	70(11)	64(13)	0.51 (0.09-0.78); 21
L. Rotation	86(8)	84(7)	0.73 (0.43-0.88); 5	66(11)	65(13)	0.70 (0.37-0.87); 17
***Cervical Muscle Strength*** (***kg***·***F***)						
Flexion	10.8(4.7)	9.8(3.7)	0.85 (0.64-0.94); 4.4	12.2(6.3)	8.8(3.4)	0.54 (0.05-0.81); 8.7
Extension*	21.4(7.6)	18.1(6.5)	0.82 (0.15-0.95); 8.2	19.2(7.7)	12.4(4.9)	0.39 (−0.10-0.73); 12.5
R. Side bend*	18.3(7.1)	15.9(4.2)	0.67 (0.33-0.86); 9.5	15.1(4.8)	14.0(3.9)	0.56 (0.22-0.82); 7.2
L. Side bend*	18.5(6.9)	14.3(4.7)	0.72 (0.01-0.91); 9.0	14.0(5.2)	12.3(3.9)	0.72 (0.37-0.89); 6.3
***Cervical Muscle Endurance*** (***seconds***)						
Flexion	33.9(24.4)	39.0(25.8)	0.72 (0.42-0.88); 34.1	29.0(14.6)	28.5(16.5)	0.40 (−0.07-0.72); 27.8
Extension	229.9(109.1)	231.8(68.3)	0.03 (−0.50-0.52); 178.3	143.8(105.6)	147.6(96.4)	0.83 (0.61-0.93); 110.2
***Scapulothoracic Muscle Strength*** (***kg***·***F***)						
R. Middle Trapezius	14.0(4.9)	13.3(4.2)	0.88 (0.71-0.95); 4.2	11.7(4.6)	10.4(2.5)	0.56 (0.18-0.80); 6.1
L. Middle Trapezius	13.9(5.3)	12.9(4.0)	0.73 (0.44-0.88); 6.3	11.6(5.1)	10.0(1.8)	0.37 (−0.06-0.69); 7.0
R. Rhomboid	19.3(8.2)	17.2(7.1)	0.88 (0.62-0.96); 7.2	15.0(5.9)	12.7(4.5)	0.59 (0.21-0.82); 8.4
L. Rhomboid	20.0(8.7)	15.7(4.9)	0.58 (0.11-0.82); 11.6	14.7(5.8)	11.7(3.8)	0.33 (−0.07-0.66); 9.2
R. Lower Trapezius	12.4(5.0)	12.0(3.0)	0.63 (0.27-0.84); 6.3	10.3(3.6)	10.2(2.8)	0.78 (0.51-0.91); 3.9
L. Lower Trapezius	12.1(4.7)	12.2(3.3)	0.73 (0.44-0.89); 5.4	11.0(4.4)	9.6(2.5)	0.65 (0.28-0.85); 5.3
***Scapulothoracic Muscle Length*** (***cm***)						
R. Latissimus Dorsi*	6.4(5.2)	6.9(5.1)	0.91(0.78-0.96); 4.2	5.1(3.5)	10.7(4.0)	0.19 (−0.11-0.53); 7.6
L. Latissimus Dorsi*	6.1(5.0)	6.9(5.6)	0.93 (0.82-0.97); 3.8	5.6(3.6)	9.6(3.9)	0.23 (−0.11-0.57); 7.4
R. Pectoralis Minor	4.9(1.5)	4.9(1.5)	0.71 (0.40-0.87); 2.1	5.7(1.4)	5.9(1.1)	0.82 (0.59-0.93); 1.4
L. Pectoralis Minor	4.5(0.9)	4.6(0.8)	0.40 (−0.05-0.72); 1.6	5.1(1.2)	5.5(0.9)	0.68 (0.34-0.86); 1.6

ICC = Intraclass correlation coefficient; 95% CI = 95% confidence interval; MDC = Minimum Detectable Change; R=right; L=left; Ave= Average measurement across subjects for each rater; SD=Standard Deviation. * indicates measures demonstrating significant rater effect, p < 0.05.

**Figure 5:**
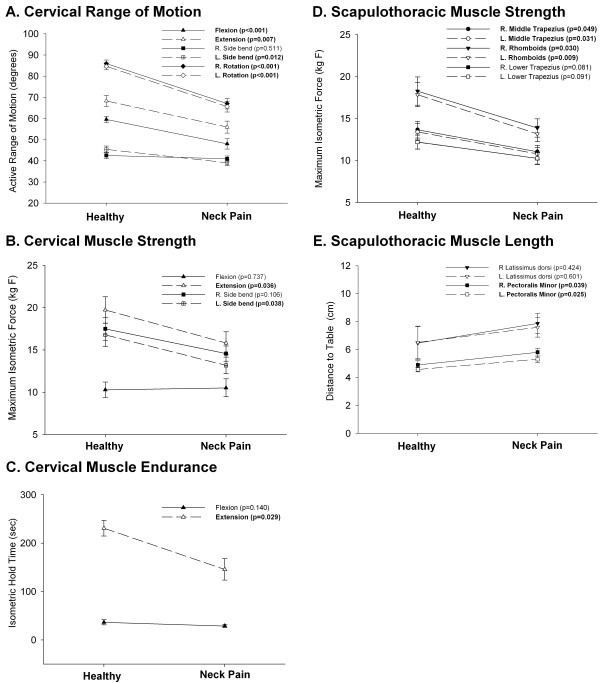
**Mean** (**SE**) **of cervical and scapulothoracic impairment measures for neck pain and healthy control groups**. **Significant differences between groups** (**bold**) **are indicated by p values in legend.**

Group differences for cervical and scapulothoracic impairment measures are illustrated in Figure 5. The neck pain group had significantly less cervical range of motion in all directions compared to the healthy group (P ≤ 0.012), with the exception of side bending toward the right (P = 0.511). The strength (P < 0.036) and endurance (P < 0.029) of isometric cervical extension was also significantly lower in the neck pain group, as was the strength of cervical side bending toward the left (P < 0.038). The neck pain group exhibited significantly lower strength of the rhomboids and middle trapezius muscles bilaterally (P ≤ 0.049), with a trend toward reduced strength of the lower trapezius (P ≤ 0.091). Finally, pectoralis minor muscle length was significantly reduced on both sides (p ≤ 0.039) in the neck pain group compared to healthy individuals.

## Discussion

### Reliability and minimum detectable change of impairment measures

The majority of impairment measures examined in this study were found to have moderate to almost perfect agreement between raters. Exceptions to this observation, which demonstrated no significant agreement between raters in participants with chronic neck pain, included strength of the cervical extensors and left (i.e., non-dominant) scapulothoracic muscles, endurance of the cervical extensors, and latissimus dorsi muscle length. In contrast to the neck pain group, all measures exhibited significant agreement between raters in healthy individuals except for endurance of the cervical flexors and left pectoralis minor length. The larger number of test items with poor reliability among patients with neck pain may reflect true variations in test performance across days due to changes in chronic symptom severity. Similarly, the larger number of test items with poor reliability on the non-dominant side for both study populations may reflect true variations in maximal performance of the less active limb. In accordance with the poor reliability of these test items, their high MDC values are of limited use to detect clinically meaningful changes across time.

Our reliability coefficients and MDC values are generally in agreement with previous reports. Cleland et al [8] demonstrated substantial agreement (ICC = 0.66-0.78) for cervical range of motion assessed by different raters on the same day for a mixed sample of patients with acute and chronic neck pain. Similar to our results, MDC values for cervical range of motion have been reported in previous literature to range from 10–19 degrees [8,10]. Together, these findings indicate that substantial changes in cervical range of motion are necessary to detect clinically significant improvement across time. Our reliability coefficients for cervical range of motion in healthy individuals are slightly lower than those reported in a recent meta-analysis [7]. However, previous studies evaluated reliability over a shorter time interval, which may not adequately reflect the true day-to-day variability of these measures in the healthy population. The inter-rater reliability for cervical muscle strength assessed using handheld dynamometry has been described as “doubtful” due to the limited number of existing studies with poorly described methodology regarding blinding procedures, examiner qualifications, and duration of the test-retest interval [5]. The present study demonstrates substantial to almost perfect agreement for cervical strength measures in healthy individuals and fair to substantial agreement for individuals with chronic neck pain, using a clearly defined study methodology and test procedures (Figure 1) that are reliable when implemented by both novice and expert clinicians.

Our findings are generally consistent with previous reports of moderate to substantial reliability of cervical flexor endurance in neck pain and healthy populations [8,11], [23,24]. However, only one study has examined the reliability of neck extensor endurance using comparable methods. These authors reported similar reliability coefficients among a smaller sample of patients with postural neck pain, but lesser MDC values for both flexor (17.8s) and extensor (71.3s) endurance tests [20]. In contrast to high measurement reliability for cervical extensor endurance in our neck pain sample, reliability was poor for the control group.

We are aware of only one previous report of inter-rater reliability for scapulothoracic muscle length and strength measures in individuals with neck pain [8]. This study reported substantial to almost perfect agreement (к = 0.69 to 0.81) for rhomboid strength and latissimus dorsi and pectoralis minor length measures, and poor agreement for middle and lower trapezius strength measures (к = −0.04 to −0.07). However, these outcomes were dichotomized based on whether the muscle was judged to be normal or restricted, and the results are not directly comparable to the present study in which muscle length and strength were quantified on a continuous scale. MDC values for scapulothoracic muscle length and strength measures have not been previously reported for individuals with chronic neck pain.

### Group differences in impairment measures

Individuals with chronic neck pain exhibited multiple impairments in cervical and scapulothoracic muscle performance compared to healthy participants, supporting the ability of these measures to differentiate among individuals with and without neck pain. Group averages for cervical range of motion indicated a significant decrease in cervical range of motion for the neck pain group in all directions except side bending toward the right. This finding is consistent with previous reports of impaired cervical range of motion among individuals with neck pain [25,26]. One explanation for restricted side bending range of motion toward the non-dominant, but not the dominant limb in the neck pain group may be that the dominant upper trapezius muscle is shortened in individuals with chronic neck pain. This would restrict cervical motion during movement toward the non-dominant side when the dominant trapezius muscle is stretched. Although trapezius muscle length was not measured in the present study, a high prevalence of upper trapezius length restrictions (right > left) among patients with neck pain has been reported previously [8]. Interestingly, asymmetrical adaptations of upper trapezius muscle length have been proposed as one potential source of pain in individuals with postural alignment faults [15]. Our findings also revealed bilateral shortness of the pectoralis minor muscle in patients with neck pain compared to healthy individuals. The pectoralis minor muscle has attachments on the coracoid process of the scapula and the anterior ribs, and functions to elevate and anteriorly tilt the scapula [27]. A position of greater scapular elevation could alter postural mechanics to facilitate adaptive shortening of other scapular elevators such as the upper trapezius and levator scapulae muscles, which are common sites of local trigger points among individuals with non-traumatic neck pain [15].

Our measures revealed significant impairments of cervical extension strength and endurance in the neck pain group that were apparent despite high measurement variability across days. The strength of cervical side bending toward the left (i.e., non-dominant) limb was also impaired in the neck pain group. Previous studies have reported weakness in both cervical extensor and flexor musculature among individuals with non-specific neck pain [28], as well as unilateral side bending strength deficits toward the left among symptomatic fighter pilots [29]. Although we did not observe the same deficits in cervical flexion strength reported in these studies, our findings are consistent with other reports of intact strength and endurance of the cervical flexors and reduced strength and endurance of the cervical extensors among individuals with chronic neck pain [28,30]. Thus, our observations confirm the presence of strength and endurance deficits for the cervical extensors, but not the cervical flexors, in a larger sample of patients with chronic neck pain than examined in previous studies.

Compared to healthy individuals, the neck pain group demonstrated significant bilateral weakness of the rhomboid and middle trapezius muscles, with a trend toward bilateral weakness of the lower trapezius. These scapulothoracic muscle groups are involved in postural stability and help reduce biomechanical loading of the cervicoscapular muscles [14]. It has been hypothesized that imbalances in scapulothoracic muscle performance, such as increased stiffness and overuse of the upper trapezius combined with weakness and inhibition of the middle and lower trapezius, may contribute to chronic pain syndromes [14]. Although postural correction and strengthening of the scapulothoracic muscles have been advocated for the prevention and treatment of neck pain in clinical practice [15], this study provides the first evidence of strength deficits in the middle trapezius and rhomboid muscles in patients with chronic neck pain compared to healthy individuals. Together with a recent study demonstrating lower trapezius strength deficits in patients with unilateral neck pain [16], these findings suggest a need for future clinical trials to determine the efficacy of scapulothoracic muscle strengthening in the management of chronic neck pain.

### Study limitations

Several important limitations of the present study must be recognized. First, the confidence intervals around our point estimates for inter-rater reliability varied widely, which may have contributed to an overestimation of MDC values. Although a larger sample size could have reduced measurement variability, our study sample is comparable or larger in size than most previous studies and our findings are consistent with other reports of large uncertainty in the reliability of clinical outcome measures for neck pain [8]. Additionally, this is one of the first studies to examine the between-day reliability of chronic symptoms which are more likely than acute symptoms to fluctuate over time.

Second, we did not record pain levels during testing and therefore cannot determine to what extent changes in the location or severity of symptoms affected test performance among the neck pain group. Changes in pain severity across the different test days and examination items likely contributed to greater measurement variability, and also may have underestimated the maximum performance capacity of individuals with neck pain compared to pain-free individuals; however, the magnitude of these effects is currently unknown. Although changes in pain severity were not systematically recorded during testing, the study examiners were not blinded to group status so that any worsening of symptoms that may have occurred during the examination could be monitored as a safety precaution. A lack of blinding could have biased our results, however, this threat was minimized by the use of standardized and objective measurement techniques. All participants were able to tolerate the full 30-minute examination without a significant increase in symptoms, although some participants with chronic neck pain reported delayed onset muscle soreness lasting up to 48 hours after the examination.

Finally, the majority of our study population comprised young to middle-aged individuals with mild to moderate levels of disability associated with chronic pain. Therefore, findings from this investigation may not generalize to older populations, or to patients with acute symptoms or more severe neck pain and disability.

### Future directions

Findings from the present study establish the measurement reliability and MDC of cervicothoracic impairments that differ between individuals with and without neck pain. Future longitudinal studies are needed to assess whether these impairment measures are responsive to change following interventions for neck pain, and to what extent improvements in cervicothoracic impairments are associated with recovery of pain, function, and disability.

## Conclusions

Our findings demonstrate that inclinometry and hand held dynamometry can provide objective and reliable measurements of cervical and scapulothoracic muscle performance as commonly applied in clinical practice where impairments are often assessed on different days, and by therapists with different levels of clinical experience. These tools are relatively inexpensive, efficient, and safe for clinical use. We have further documented the minimum detectable change necessary to identify significant clinical improvement in these measures across time, thereby providing a valuable alternative to more subjective assessment methods that lack sensitivity to change. Finally, we have identified which measures are likely to reveal impairments in patients with neck pain compared to healthy individuals, and therefore may be considered as potential targets for the assessment and management of neck related impairments.

## Competing interest

The authors declare that there is no conflict of interest.

## Authors' contributions

BS participated in development of the protocol, performed data collection, data analysis and interpretation, and preparation of the manuscript CJ participated in development of the protocol, performed data collection, reviewed data analysis and interpretation, and revised the manuscript DCE participated in development of the protocol, reviewed data collection methods, participated in data analysis and interpretation, and revised the manuscript. KSM participated in development of the protocol, reviewed data collection methods, reviewed data analysis and interpretation, and revised the manuscript. All authors read and gave final approval for this document.

## Pre-publication history

The pre-publication history for this paper can be accessed here:

http://www.biomedcentral.com/1471-2474/13/215/prepub
